# A Concert between Biology and Biomechanics: The Influence of the Mechanical Environment on Bone Healing

**DOI:** 10.3389/fphys.2016.00678

**Published:** 2017-01-24

**Authors:** Vaida Glatt, Christopher H. Evans, Kevin Tetsworth

**Affiliations:** ^1^Department of Orthopaedic Surgery, University of Texas Health Science Center San AntonioSan Antonio, TX, USA; ^2^Orthopaedic Research Centre of AustraliaBrisbane, QLD, Australia; ^3^Rehabilitation Medicine Research Center, Mayo ClinicRochester, NY, USA; ^4^Department of Orthopaedic Surgery, Royal Brisbane and Women's HospitalHerston, QLD, Australia

**Keywords:** bone repair/regeneration, dynamyzation/reverse dynamization, mechanical environment, fixation stability, large bone defects, fracture fixation, external/internal fracture fixation, bone healing/biomechanics

## Abstract

In order to achieve consistent and predictable fracture healing, a broad spectrum of growth factors are required to interact with one another in a highly organized response. Critically important, the mechanical environment around the fracture site will significantly influence the way bone heals, or if it heals at all. The role of the various biological factors, the timing, and spatial relationship of their introduction, and how the mechanical environment orchestrates this activity, are all crucial aspects to consider. This review will synthesize decades of work and the acquired knowledge that has been used to develop new treatments and technologies for the regeneration and healing of bone. Moreover, it will discuss the current state of the art in experimental and clinical studies concerning the application of these mechano-biological principles to enhance bone healing, by controlling the mechanical environment under which bone regeneration takes place. This includes everything from the basic principles of fracture healing, to the influence of mechanical forces on bone regeneration, and how this knowledge has influenced current clinical practice. Finally, it will examine the efforts now being made for the integration of this research together with the findings of complementary studies in biology, tissue engineering, and regenerative medicine. By bringing together these diverse disciplines in a cohesive manner, the potential exists to enhance fracture healing and ultimately improve clinical outcomes.

## Introduction

Fracture healing generally occurs on a routine basis and is therefore easily dismissed and often overlooked, but the process is no less remarkable. This involves a spectrum of growth factors and other stimuli interacting with one another in an organized response, and is in many ways analogous to the performance of a symphony. When an orchestra appears in concert, many musicians playing different types of instruments can create an auditory masterpiece through a carefully coordinated, and exquisitely timed performance. The same is true with fracture healing, where a wide variety of biological factors and their products must also perform in a highly coordinated fashion. To achieve the desired outcome, each individual component must fulfill a specific and equally important function. The role of the various biological factors, the timing and spatial relationship of their introduction, and how the mechanical environment influences this activity are all critical aspects to consider. The best results require the concerted activity of each modulator in the correct amounts at the proper times, many working in harmony with other factors and their products to produce robust callus resulting in fracture union. The critical role of coordinating the complex interaction of factors characteristic of this biological orchestra is filled by the mechanical environment, and one could consider this the conductor of fracture healing. Most significantly, understanding how this process normally occurs is vitally important in managing those instances when the performance is sub-optimal and a fracture fails to unite.

**Figure 1:**
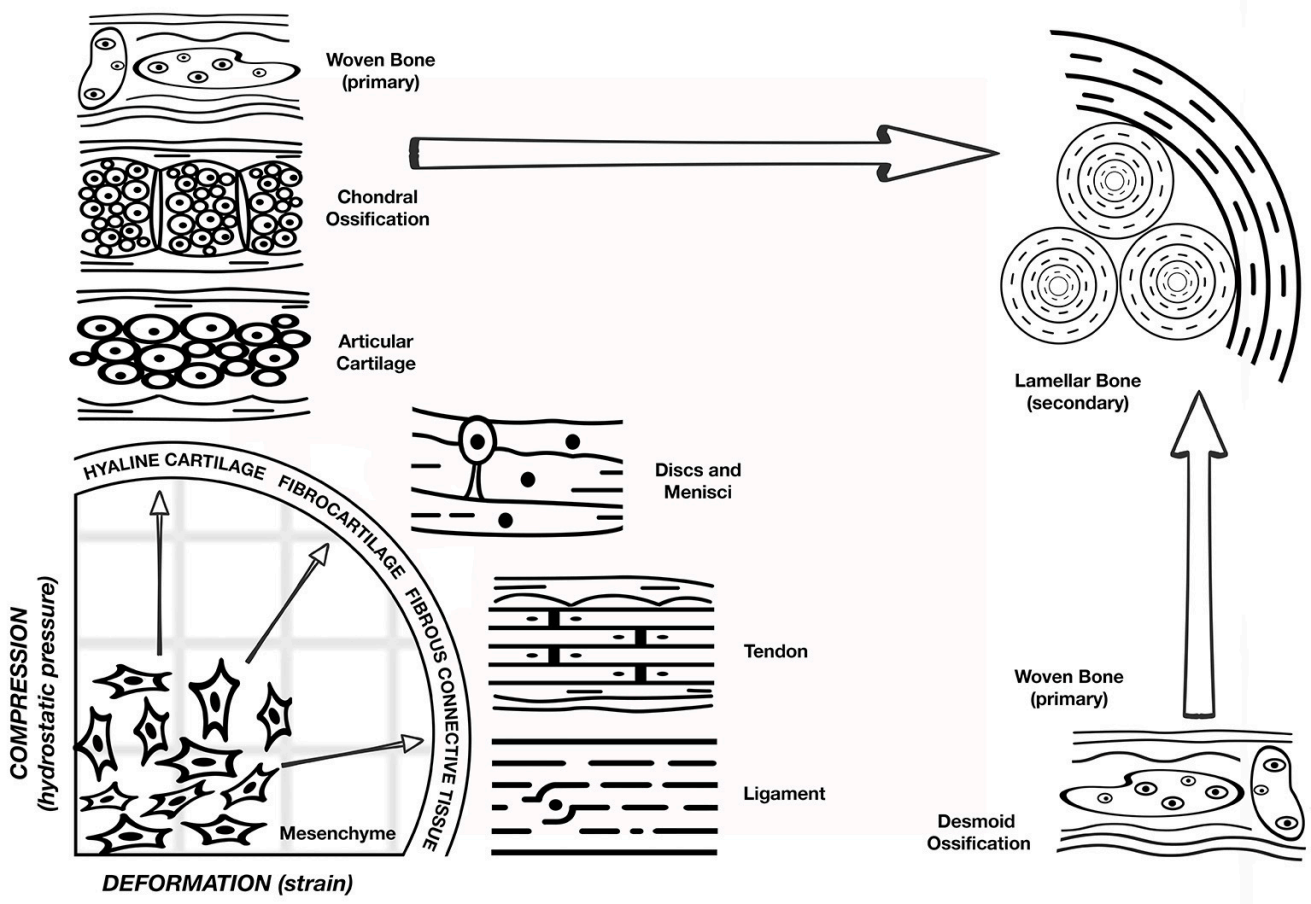
**Pauwel's concept of tissue differentiation: a schematic representation of the hypothesized influence of the biophysical stimuli on tissue phenotype**. Deformation of shape strain is on the x axis and the hydrostatic compression is on the y axis. A combination of these stimuli will act on the multipotent progenitor cells leading to hyaline cartilage, fibrocartilage, or fibrous connective tissue as shown on the quadrant perimeter. Depending on the response of the mechanical environment to the presence of these tissues, osteoblast proliferation and ossification can occur. However, bone regeneration occurs only after stabilization of the mechanical environment by the formation of soft tissue. (Figure adapted from Weinans and Prendergast, 1996).

While the biological basis of bone healing has been well studied, less is known about the mechanical factors that play unique and key roles in the success of the repair process. It has been known since the time of Wolff (1892) that bone is exquisitely responsive to its mechanical environment. The general principal of Wolff's Law (1892) states that skeletal elements are strategically placed to optimize strength in relation to the distribution of applied loading, and that the mass of the skeletal elements is directly related to the magnitude of the applied loads (Wolff, 1986). Even earlier, Roux (1881) proposed the idea that cells within tissues engage in “a competition for the functional stimulus,” and it is this competition that determines cell survivorship and therefore tissue phenotype (Roux, 1881). He hypothesized that the mechanical environment has a very specific relationship with phenotype, where tension results in fibrous connective tissue, shear forms cartilage, and compression produces bone. Roux called this phenomenon *Entwicklungsmechanik* (developmental mechanics). He postulated that structural adaptation of tissue to mechanical loads is a direct consequence of competition between cells. Years later, the discoveries of Pauwels refined the concept that the differentiation of mesenchymal progenitor cells into chondrocytes and osteoblasts is regulated by mechanical forces (Figure 1). Since then, many researchers have investigated this notion and have shown the differential effects of the type and magnitude of mechanical forces on tissue formation (Pauwels, 1960).

This review discusses the current state of the art concerning the application of these mechano-biological principles to enhance bone healing, by controlling the mechanical environment under which bone regeneration takes place. PubMed served as the main search engine to identify relevant articles. Keyword combinations included variations of the following phrases: mechanical environment; fracture healing; bone healing; fixation stability; dynamization; large bone defects; tissue regeneration; animal models; growth factors; bone morphogenetic proteins; mechanical signals; mesenchymal stem cells; intramedullary nails; external fixators; internal fixators; orthopedics; fracture fixation.

## Basic principles

Bone is a mechanosensitive organ and has the ability to respond to the mechanical stimuli placed upon it. After skeletal maturation, it continues to remodel throughout life and is reshaped in response to the mechanical forces acting upon it. The role of physical factors on bone regeneration cannot be addressed without mention of mechano-biology, which is the study of how mechanical and physical conditions regulate biological processes (Carter et al., 1998). Much of our present day understanding about the effects of mechanical forces on tissue differentiation comes from Pauwels (1960). Building on the earlier developmental mechanics of Roux, he was one of the first to recognize that physical factors cause stress and deformation of mesenchymal progenitors, including what we now refer to as mesenchymal stem cells (MSCs), and that such mechanical stimuli could determine cell fate. Pauwels (1960) compared the mechanical environment of cells in a fracture callus with fracture repair patterns, and proposed that deformation (strain) of shape is a specific stimulus for the formation of collagenous fibers, and hydrostatic compression is the specific stimulus for the formation of cartilage. Osteogenesis, however, requires that the mechanical environment first becomes stabilized by the presence of fibrous tissue (Perren and Cordey, 1980). Therefore, Pauwels' hypothesis, as shown in Figure 1, is that the mechanical environment determines tissue phenotype. Carter (Carter et al., 1988) further developed this concept as a function of shear and hydrostatic stress, called the osteogenic index.

It is possible to use such concepts to help explain the cellular events occurring during fracture healing. A study by Lacroix and Prendergast showed that local tissue stresses and strains not only alter the pressure on bone cells, but also influence cell differentiation (Lacroix and Prendergast, 2002). For instance, in the fracture gap after fixation hydrostatic pressure is relatively low, but intrafragmentary strains (IFS) are relatively high. However, when callus becomes larger the stiffness in the fracture increases, while at the same time rising hydrostatic pressure decreases the matrix permeability, and this in turn reduces the shear strains in the fracture gap. In this environment, increasing numbers of chondrocytes are formed, and endochondral ossification begins. As more collagen matrix is produced the strain declines further, more osteoblasts accumulate, and ossification predominates. It is plausible that mesenchymal progenitors cannot differentiate into bone cells or chondrocytes unless a suitable biophysical environment for tissue differentiation is present. Therefore, for bone healing to occur the fracture environment has to be exposed to the strain rates that elicit bridging callus, increasing collagen synthesis, and rising hydrostatic pressures.

In fact, it is widely accepted that the molecular and cellular responses to the spatiotemporally specific mechanical stimuli applied at the osteotomy site will guide the migration, proliferation, and differentiation of endogenous skeletal progenitor cells. Collaborative research efforts in the fields of molecular and cell biology, bioengineering, and the material sciences expanded our understanding of the molecular pathways involved in the process of bone regeneration including mechanotransduction, progenitor cells homing and differentiation, and neovascularization. From these studies specific molecular pathways have been identified as being the critical contributors to the tissue regeneration process including those involving growth factors, morphogens, cytokines, signal transduction, and epigenetic changes, among others (Carter et al., 1998; Kelly and Jacobs, 2010; Wang et al., 2011, 2012; Thompson et al., 2012). However, exactly how the bone cell population is influenced by the mechanical environment when responding to mechanical signals to regenerate and remodel a successful structure is still uncertain. Nevertheless, it is certain that it occurs at various levels including organ, tissue, cellular, and molecular contributions. For instance, exposure to the physical factors at the organ level such as force, displacement, and deformation at the fracture site will have an effect on the behavior of bone cells, which in turn will determine the type of tissue formed. At the tissue level, the mechanical stimulus characterized by stress and strain parameters will determine different patterns of tissue formation. Moreover, the influence of mechanical signals at the cellular level would take into account changes of cell shape, cell pressure, as well as oxygen tension, and this will determine the patterns and production of extracellular matrix components. And finally, mechanical signals sensed at the molecular level will determine changes in the cytoskeleton, resulting in intracellular signaling that promotes specific cell activities. While the specifics of these processes are beyond the scope of this review article, details can be found in the review articles by Rubin, Chen, and many others (Rubin and Hausman, 1988; Uhthoff et al., 1994; Carter et al., 1998; Morgan et al., 2008; Huang and Ogawa, 2010; Kelly and Jacobs, 2010; Thompson et al., 2012).

Understanding the nature of these mechanical cues, and the biological responses to them at various levels is very important as this will determine the quality, the type of tissue formed, and the rate of the healing process. For example, at the organ level, instability of fracture fixation will result in fibrous and cartilaginous tissue formation, that will most likely lead to a delayed union or a non-union. At the cellular level, excessive mechanical stimulation will influence cells to proliferate and differentiate toward specific lineages, and this will be dependent upon the flexibility of the fracture fixation. Likewise, at the molecular level, specific pathways will be activated to produce a specific type of tissue, reflecting mechanical cues at the organ level. The aforementioned clearly shows how a stimulus initiated at the organ level (fixation stability) would lead to changes at the molecular level which determine the rate, type, and the quality of the tissue regeneration process. Numerous studies have also shown that bone regeneration and remodeling are sensitive to changes in strain magnitude (Rubin and Lanyon, 1984), the number of loading cycles (Rubin and Lanyon, 1984), the distribution of loading, and the rate of strain. Findings from these studies suggest that cells respond not only to mechanical deformation (hydrostatic pressure) (Qin et al., 2003) but also to fluid flow (Meinel et al., 2004; Jekir and Donahue, 2009; Stiehler et al., 2009; Qin and Hu, 2014), and in an *in vivo* setting both of these biomechanical stimuli are present (Pacicca et al., 2002; Huang and Ogawa, 2010). Furthermore, the cellular response from biomechanical loading is heavily dependent upon the magnitude of strain (Yanagisawa et al., 2007, 2008; Luu et al., 2009; Klein-Nulend et al., 2013; Uzer et al., 2015), the type of loading conditions (Tanno et al., 2003; Haasper et al., 2008), and on the differentiation stage of the progenitor cells (Weyts et al., 2003; Jansen et al., 2004).

## Influence of mechanical forces on bone healing: fractures, osteotomies, and segmental defects

Many articles describe the effects of mechanical stimulation on the healing of fractures in both laboratory animals and human subjects. The work of Perren, Carter, Claes, Kenwright, Goodship, and others have shown convincingly that manipulation of the ambient mechanical forces around a fracture site can determine whether a fracture heals or not, and will determine both the rate of healing and whether healing occurs via intramembranous or endochondral pathways (Carter and Wong, 1988a,b; Grundnes and Reikerås, 1993; Utvåg and Reikerås, 1998; Augat et al., 2005). The mechanical environment is determined by the stiffness of fracture fixation and weight bearing; if fixation is either too flexible or too rigid the healing might fail. For instance, as long as high strain forces exist at a fracture site (inadequate stability), fibrous tissue will remain, and stabilizing bony callus will be unable to form. If such conditions continue for long enough, a fibrous non-union will result (Carter et al., 1988; Claes et al., 1997). Earlier studies agreed that rigid fixation provided the best clinical outcome, and this has led to the wide use of nailing and plating to provide rigid internal fixation (Grundnes and Reikerås, 1993). However, some studies have also demonstrated that excessively rigid fixation may paradoxically impair fracture healing (Goodship and Kenwright, 1985; Aalto et al., 1987; Chao et al., 1989). Major concerns raised are the possible inhibition of external callus formation, maintenance of a fracture gap aggravated by bone-end resorption, and the excessive protection of the healing bone from normal stresses (stress shielding), producing adverse remodeling. Consequently, based on the results from these studies, many mechanical factors have been identified as having an influence on fracture healing, including the magnitude and direction of interfragmentary movement (IFM), the type of fracture, and fracture geometry (Aro and Chao, 1993; Augat et al., 1998, 2003).

IFM is the movement occurring between fracture fragments in response to physiological and external loading of the fractured limb. When the fractured bone is loaded, the fracture fragments displace relative to each other, producing a multiaxial strain that varies spatially throughout the fracture gap (Perren and Cordey, 1980). Depending on the external load phase and muscle activity, any combination of axial forces and bending or torsional moments can occur. There are two main factors that influence IFM; the rigidity of the implant used to stabilize the fracture and the surface area of the fracture fragments, which determine the tissue strain and the cellular reaction in the fracture gap. The amplitude and the direction of IFMs in the fracture gap also depend upon the load applied through weight bearing, muscle forces, and the stiffness of the chosen device (Klein et al., 2003; Augat et al., 2005; Epari et al., 2006). Strain resulting from IFM is distributed over the fracture surface and will differ depending on the fracture geometry. For instance, comminuted fractures will be able to tolerate relatively greater motion since the strain is applied over a larger surface area of fracture fragments. The size of callus formed during the healing process will depend on the magnitude of IFM (McKibbin, 1978; Goodship and Kenwright, 1985; Claes et al., 1997, 1998; Wu, 1997). If IFM exceeds a critical level, the blood vessels formed at the fracture site will be subjected to repeated disruption and would not become established, preventing the development of stable tissues (Carter et al., 1988; Claes et al., 1997). For example, a certain amount of mechanical instability leads to greater IFM with the formation of cartilage, and thus to endochondral healing by which the majority of fractures heal (Augat et al., 2005). Interfragmentary strain (IFS) did not influence healing of osteotomy gaps of 1 mm or smaller in a sheep model. However, decreased bone formation and inferior mechanical properties of healed bone were evident when larger IFS were applied to 2 and 6 mm fractures gaps (Augat et al., 1998). This leads to the conclusion that larger gaps require stable fixation that is still flexible enough to stimulate initial callus formation. This is preferable to fixation that is very unstable or too rigid. Moreover, both the quality of the tissue along the osteotomy line and the width of the osteotomy gap help to determine the mechanical quality of the healed bone (Augat et al., 1998).

Numerous experimental studies have been conducted to isolate the type of loading (axial, shear, torsion) and the effect it has on bone healing (Yamagishi and Yoshimura, 1955; Kenwright and Goodship, 1989; Park et al., 1999; Duda et al., 2002; Augat et al., 2003; Klein et al., 2004; Bishop et al., 2006). Moderate compressive axial IFMs enhanced periosteal callus formation and increased the rate of fracture healing in both animal models (Yamagishi and Yoshimura, 1955; Kenwright and Goodship, 1989; Klein et al., 2003; Augat et al., 2005) and clinical studies (Noordeen et al., 1995). However, both shear and tensile IFMs of similar magnitude appeared to inhibit fracture repair (Yamagishi and Yoshimura, 1955; Augat et al., 2003; Klein et al., 2004). In fact, shear movement considerably delayed the healing of experimental fractures, and in most instances produced only partial bridging and less persiosteal callus formation, resulting in significantly delayed healing (Augat et al., 2003, 2005). Although these studies clearly demonstrate the impact of IFM on bone healing, more research remains to be done to understand fully the biological processes involved.

Many computational models have also been developed to study the effect of the mechanical environment on fracture healing. These again confirm that the stiffness of fixation influences fracture healing. Gómez-Benito et al. (2006) developed a finite element model of a simple transverse mid-diaphyseal fracture of an ovine metatarsus fixed with bilateral external fixators of three different stiffnesses. Their calculations predicted that a low stiffness external fixator would delay fracture healing and cause a larger callus than a more rigid external fixator. Carter et al. (1998) examined the importance of cyclic motion and local stresses and strains on bone tissue formation. They confirmed the basic mechanobiologic concepts that bone formation is promoted in the areas of low to moderate tensile strain, fibrous tissue is promoted in the areas of moderate to high tensile strains, and chondrogenesis is promoted in areas of hydrostatic compressive stress (pressure). On the other hand, Claes et al. (1998), using a sheep model, investigated the influence of the osteotomy gap size and IFM on fracture healing. They hypothesized that gap size and the amount of strain and hydrostatic pressure along the calcified surface in the fracture gap are the fundamental mechanical factors involved in bone healing. They proposed that intramembranous bone formation would occur for strains smaller than approximately 5% and hydrostatic pressure of no more than 0.15 MPa. On the contrary, strains up to 15% and hydrostatic pressure of more than 0.15 MPa would stimulate endochondral ossification. As expected, they found that there was a significant decrease in the rate of healing with an increasing osteotomy gap size. Furthermore, they found that a 2 mm gap led to greater IFM, more periosteal callus, and an increased amount of connective tissue in the fracture gap compared to osteotomies 1 mm or smaller. In fact, large critical sized gaps of 6 mm never healed during a period of 9 weeks, and they mainly produced fibrous connective tissue in the osteotomy gap regardless of the amount of IFM.

Kenwright and Gardner (1998) summarized studies that measured interfragmentary displacement in six degrees of freedom throughout healing in patients with tibial diaphyseal fractures treated by external fixation, and developed a finite element analysis model of healing tibial fractures. The model predicted that tissue damage might occur in the later (hard callus) phase of healing, even while the fixation device is still in place, because of very high stresses and strains. This study also indicated that the mechanical environment should be better controlled to provide amplitudes of movement in the first weeks of healing, and that the rigidity of fixation should then be increased to optimize the fracture healing process until the fixator is removed.

## Dynamization

Many authors have suggested that the delayed introduction of controlled motion (“dynamization”) as healing progresses may lead to faster maturation of bone (De Bastiani et al., 1984; Wu, 1997; Arazi et al., 2002), but this remains controversial and has not greatly influenced clinical practice (Gorman et al., 2005; Tigani et al., 2005; Claes et al., 2009). Dynamization is a word used when the IFM is increased by changing from a stable fixation to a more flexible fixation. It is often used when an implant allows axial shortening of a bone through a telescoping mechanism incorporated into the fixation device (De Bastiani et al., 1984). This regimen of treatment was only implemented after recognizing that IFM between the fracture ends is beneficial to the healing process, provided it is controlled. External fixation devices can readily be changed from a stable to a dynamic configuration, thereby allowing for more axial movement between the fracture fragments. Surprisingly, there have not been many fracture fixation devices specifically developed that actively incorporate dynamization in the clinical treatment of fractures. At this time it remains unclear when during the healing process dynamization should be applied, and whether it helps fractures repair more efficiently. For example, there have been a few clinical studies that have attempted to determine the optimal axial IFM or the effect of dynamization at the various stages of fracture repair, and it is unclear whether this accelerates bone repair in an efficient and timely manner (Marsh et al., 1991; Siguier et al., 1995; Domb et al., 2002). There are no clinical studies to our knowledge that have attempted to determine the effects of dynamization on the healing of large segmental bone defects.

The results of animal studies on the effects of dynamization on bone healing are inconsistent. A study by Larsson et al. (2001) investigated the effect of early axial dynamization (1 week post-op) on tibial bone healing in a canine model. They used a rigid external fixator to stabilize a 2 mm transverse osteotomy on both tibias of each dog to allow paired comparison of the results. They found that early dynamization resulted in accelerated callus formation and maturation, with increased remodeling of endosteal and periosteal callus tissue. Moreover, the dynamized side showed significantly higher torsional stiffness after 5 weeks of treatment than did the controls. A study by Aro and Chao (1993) also investigated bone healing patterns affected by loading, fracture fragment stability, fracture type, and fracture compression in a canine osteotomy model. This study had three different groups: transverse and oblique fractures were fixed with a rigid unilateral external fixator, with bone fragments separated by a distance of 1 mm, 2 mm, or in contact. Dynamization with uniform axial loading and motion was performed at either 2 or 4 weeks. They found that, for a given rigidity of external fixation, the amount of physiological stress and the presence of a significant gap proved to be the most significant factors in determining the pattern of fracture repair. Motion with loading tended to promote external callus maturation and secondary bone healing.

On the contrary, two studies using a rat model found that neither early nor late dynamization was beneficial to fracture healing. These two studies used very different fixation devices, including a rigid IM nail (Utvag et al., 2001) and an external fixator (Claes et al., 2009). Both groups found that dynamization increased callus formation, but reduced the quality of healed bone. These findings are not surprising, given that the evidence in the literature shows that increased IFM resulting from a flexible fixation device leads to greater callus formation, a prolonged chondral phase, and delayed healing by disrupting the vascular supply needed for bone tissue to repair and remodel (Rand et al., 1981; Ozaki et al., 2000). In fact, a study by Cullinane et al. (2002) attempted to influence cell fate decisions through precisely controlled motion using a custom made external fixator that introduced IFM bending strain. This was compared to a rigidly fixed segmental defect. The results of this study showed that they were able to direct formation of cartilage and bone during fracture repair by inducing controlled motion. They also demonstrated that the spatial organization of the collagen fiber architecture within the newly formed tissue was influenced by the local mechanical environment, and noted that bone did not form when the IFM was too high.

These clinical (Wiss et al., 1986; Brumback et al., 1988; Meléndez and Colón, 1989) and experimental (Wolf et al., 1998; Hente et al., 1999; Claes et al., 2009) studies have been inconclusive and contradictory, and have failed to convincingly demonstrate any benefit of dynamization to bone healing (Table 1). Yet one thing is clear, the mechanical environment surrounding the fracture gap plays a very important role and largely determines how and if the fracture will heal. Although, in principle, dynamization is a sound strategy to attempt to accelerate the healing process its main clinical drawback is the early loss of frame stability, potentially leading to delayed union, refracture, or the development of a secondary deformity. Regardless, early dynamization has shown favorable healing outcomes as long as the treatment was applied after a bridging callus has formed (Acker et al., 1985; Kempf et al., 1985; Foxworthy and Pringle, 1995; Basumallick and Bandopadhyay, 2002).

**Table 1:** **Comparison of reverse dynamization to dynamization**.

**Reverse dynamization**	**Dynamization**
Decreases IFM	Increases IFM
Early reverse dynamization accelerates union	Early dynamization leads to non-union
Late reverse dynamization of no benefit	Late dynamization of no benefit
Has not yet been the subject of any large scale study to determine if there is any clinical benefit	Limited or no proven clinical benefit despite multiple studies attempting to do so

## Reverse dynamization

**Figure 2:**
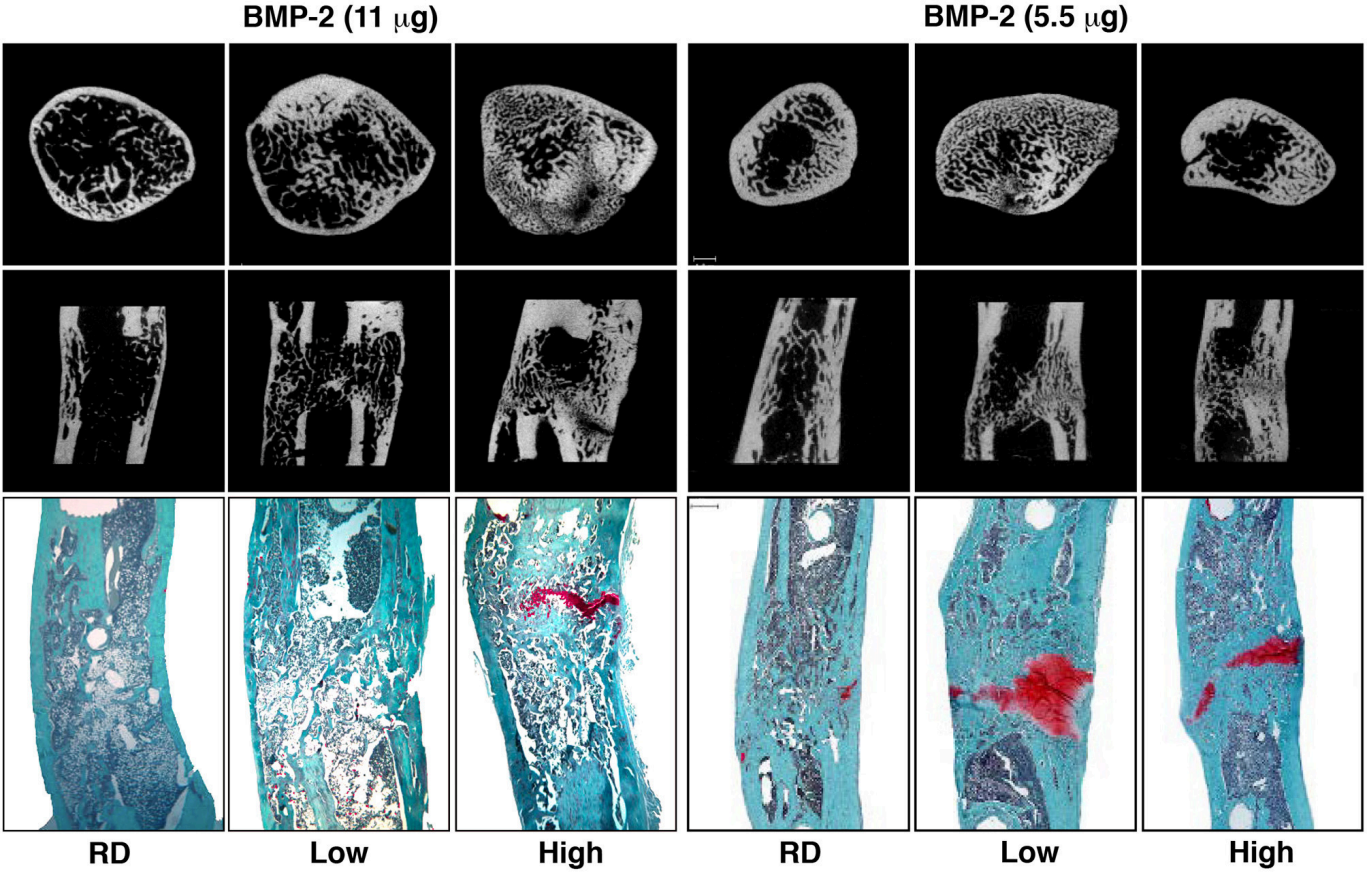
**Micro-computed tomography (μCT) and histology images illustrating healing of segmental defects with 11 and 5.5 μg doses of BMP-2, high and low stiffness external fixator groups as well as a Reverse Dynamization group (RD) after 8 weeks of treatment**. μCT images of cross-sectional distal part of the defect (top row) and coronal plane of the defect (middle row). Histological sections were stained with safranin orange-fast green (bottom row, Scale bar = 1 mm). Low = low stiffness fixator (114 N/mm); High = high stiffness fixator (254 N/mm); RD = low to high stiffness fixation at 2 weeks (from Glatt et al., 2012b, 2016a).

Based upon data from the healing of fractures and sub-critical size osteotomies, large segmental defects are subjected clinically to rigid internal fixation. However, there is little evidence to justify such an action, and few studies can be found that investigate the influence of the mechanical environment in healing large segmental bone defects. In response to this, for the past several years we have used a rat model to investigate the effects of fixator stiffness on the healing of large bone defects treated with BMP-2. We hypothesized that the healing of large, osseous defects can be enhanced by manipulating the mechanical environment as healing progresses. Specifically, we suggested that healing would be accelerated by first stabilizing the defect under conditions of low stiffness, and then imposing high stiffness once healing was underway, a strategy we call reverse dynamization. The results of this study were remarkable, confirming that the healing of large bone defects is highly responsive to the ambient mechanical environment (Figure 2), allowing the rate and quality of healing to be manipulated by altering fixation stability (Glatt et al., 2012b). Moreover, this study was the first to introduce the concept of reverse dynamization, and demonstrated its superiority as a means of accelerating the healing and maturation of bone (Figure 2). Based on these observations, a subsequent study determined whether the dose of BMP-2 could be reduced without compromising the healing process when using this enhanced mechanical environment (Glatt et al., 2016a). This study has shown that while the initial healing was slightly delayed, forming a smaller callus thoughout the healing period, the quality of healing bone was similar or slightly superior to that treated with the higher dose of BMP-2 (Figure 2). Interestingly, the same study also showed that if the dose of BMP-2 was insufficient, healing did not occur no matter which stiffness fixation device was used.

A study by Boerckel et al. (2011) reported that the application of early mechanical loading significantly inhibited vascular invasion and reduced bone formation in an 8 mm rat femoral defect when compared to stiff plate controls using two different doses of BMP-2 (0.2 and 2.5 μg). On the other hand, delaying mechanical loading by 3 weeks significantly enhanced bone formation. In a different study, using a 6 mm rat model, they demonstrated that the functional transfer of axial loads by modulation of fixation plate stiffness at 4 weeks significantly enhanced BMP-mediated large bone defect repair (Boerckel et al., 2012). Similar results to those of Boerckel et al. were reported by Claes's group who demonstrated superior results when mechanical modulation, from rigid to more flexible fixation, was applied at 3 and 4 weeks (Claes et al., 2011) after surgery compared to 1 week (Claes et al., 2009). However, for those studies a 1 mm osteotomy model stabilized with an external fixator was used. Results from these studies are not surprising and are consistent with the literature suggesting that extremely rigid fixation is detrimental to bone healing (Chao et al., 1989), and allowing increased flexibility during the later stages of healing is beneficial. Furthermore, this is attributed to the tissues occupying the fracture gap as was demonstrated by both Gardner et al. (1996) and Glatt et al. (2012a). They demonstrated if there is no material present in the fracture gap, which simulates conditions immediately post-surgery, the fixator frame provided stability at the fracture site with no contribution from the gap itself to support the fracture site. On the contrary, when they interposed material with low stiffness, which represents the early stages of healing, they found that the mechanical properties of the fixator were as important as those of the fracture material in influencing axial IFM at around 2–4 weeks post fixation. In contrast, with stiff intra-fracture materials, simulating the remodeling stage of healing, axial movement was influenced only by the stiffness of these materials, with little contribution from the fixation devices. Fracture movement arises from the combined flexibility of the fixation devices and the compliance of tissue material in the fracture gap, and is a consequence of weight-bearing and any loads applied. For dynamization implemented at the later stages of healing, accelerated bone healing is more likely a consequence of bone adaptation following Wolff's law rather than fixator dynamization itself. In fact, this hypothesis was confirmed using the reverse dynamization regimen in the same 1 mm osteotomy rat model as was used by Claes' group (Bartnikowski et al., 2016). In this study we showed that when reverse dynamization, from flexible to rigid fixation, was applied at 3 weeks the healing outcome at the end of the treatment period was the same as the dynamization regimen (Claes et al., 2011; Bartnikowski et al., 2016). However, when reverse dynamization was applied at 1 week the healing outcome was exceedingly superior to that of the dynamized group (Table 1), constant rigid and flexible fixation groups (Bartnikowski et al., 2016). On the contrary, the dynamization regimen at 1 week was detrimental to bone healing when compared to any of the other groups tested (Claes et al., 2009).

Suprisingly, only one study can be found that investigated a reverse dynamization regimen in humans. A study by Howard et al. (2013) found that allowing initial axial macromovement from 2 to 4 weeks after surgery followed by more rigid stabilization accelerated fracture healing. Faster healing was associated with a reduction in the average time to removal of the fixator in the dynamization group (~11 weeks), compared to the standard of care (~22 weeks) when treating isolated closed or open grade I tibial fractures.

Such studies are only beginning to provide knowledge about the influence of modulating the mechanical environment and the effects it has on bone repair. The data from these studies appear to suggest there is no single set of mechanical circumstances that suits all stages of fracture repair, and that healing might be enhanced by changing the stiffness of fixation systematically during different phases. Selecting the specific mechanical conditions, as well as determining the most appropriate time to modulate this environment, will be critical to achieve the optimal conditions for bone repair. This is particularly true for reverse dynamization, where initially flexible conditions are followed by more rigid fixation, as this new concept requires further investigation to better define its role clinically (Glatt et al., 2016b).

## How this knowledge has influenced clinical practice

Physicians and healers have managed fractures non-operatively for thousands of years, using splints and other simple devices. Over the course of the past century, orthopedic implants have revolutionized the treatment of bone injuries based on new discoveries combining basic and clinical research, advances in implant technology, materials science, and improved surgical techniques. The main purpose of fracture fixation is to provide more anatomical alignment to the fragments of broken bone, and to achieve sufficient mechanical stability so that the biological process of bone healing is not disturbed. The link between mechanical and biological processes of bone regeneration and repair has been investigated for many decades. Knowledge gained from empirical, experimental, and clinical studies has tremendously changed and improved how fractures are treated today. Possible fixation strategies to treat bone fractures range from splints to external and internal fixators, and the decision to use a particular device depends on the specific bone involved, the fracture location, and the type of fracture, either “closed” or “open.” The rigidity of the fixation device used determines the degree of movement of fracture ends relative to each other that occurs through weight-bearing (external loading) and muscle contractions, and will govern the formation of specific tissues in the fracture gap. The following sections will outline the types of fixation methods used for load bearing bones, and how those methods have been influenced by our greater appreciation of the contributions of mechanobiology to fracture healing.

### Splinting

Management of a wide variety of orthopedic injuries requires the use of a splint. Splinting is used for acute fractures where swelling is anticipated, or for the initial stabilization of reduced, displaced, or unstable fractures before surgical intervention. No one knows when the first splint was used, but injured limbs have been bandaged or immobilized in some fashion since ancient times. Interestingly, despite its name, Arabians were reported to be the first ones to use the technique of pouring a plaster-of-paris mixture around the injured limb. This technique was brought to the attention of European practitioners in the early nineteenth century. Malgaigne recorded in detail the various techniques of its use, but was not keen to use it himself after having problems with swelling within a rigid cast (Malgaaigne, 1859). He subsequently abandoned the technique in favor of albuminated and starched bandages, as recommended by Seutin (Browner et al., 2008).

Different types of splints are used for various circumstances; they differ in their construction and indication. For example, a long bone fracture immobilized by a splint will be subjected to an intermittent compressive axial force imposed at the fracture site as a result of muscle activity and partial weight bearing. This allows a large degree of IFM and will typically induce healing through abundant callus formation (McKibbin, 1978; Sarmiento and Latta, 1981). The greatest disadvantage of prolonged immobilization of the limb is that the patient is often confined to bedrest until the fracture heals, which is subject to risks and complications. Modern surgeons now agree this is generally contrary to the overall health of the patient, and is avoided or minimized whenever possible. Seutin should be credited as one of the first physicians to appreciate that complete immobilization of the limb should be avoided, and in the mid nineteenth century was already promoting the benefits of fracture massage and early mobilization (Browner et al., 2008). Seutin gained many followers, but others still believed that total immobilization was a better choice. However, he continued to emphasize the importance of joint motion and one of his advocates, Lucas-Championniere, later confirmed these benefits in animal experiments (Lucas-Championniere, 1881). He went so far as to recommend massage of the injured limb to encourage motion between the fragments, generating more robust callus. This controversy between mobilizers and immobilizers resulted in the development of new splinting techniques such as the Thomas splint, various designs of traction devices, and functional braces. Perkin, Russell, Dowden, and many others were major advocates of movement, both active and passive, as the most important factor for the optimal functional outcome of the involved limb (Russell, 1924; Browner et al., 2008). The widespread use of functional bracing minimized hospitalization and permitted an earlier return to work and daily activities. However, despite historical evidence demonstrating that the influence of the mechanical environment on bone healing was already recognized in the nineteenth century, the significance of its role and its potential benefits are still actively debated today.

### Open reduction internal fixation (ORIF)

Splinting, casting, and traction techniques are suitable for many fractures, and the vast majority of these will unite spontaneously when adequately reduced and immobilized. However, closed (non-operative) methods are not suitable in many circumstances, and the prolonged immobilization or bedrest often required is associated with its own set of risks and complications. Pneumonia, contractures, ulcers, and loss of motion are not uncommon; thromboembolic phenomena, including both deep vein thrombosis and pulmonary embolism, are more serious possibilities. Because of these issues, and driven by patient expectations and the economic realities of prolonged hospitalization, modern fracture care has become far more dependent on operative stabilization. Progress in medicine and surgery over the course of the past 150 years now allows surgeons to intervene in a tremendous variety of pathological conditions, both acute and chronic. Advances in metallurgy, the development of biocompatible alloys, and improvements in the production quality of implants have paralleled the evolution of surgical science over this period. These elements have all conspired to provide orthopedic surgeons with unprecedented abilities to consider operative stabilization of many fractures, for a wide variety of indications.

**Figure 3:**
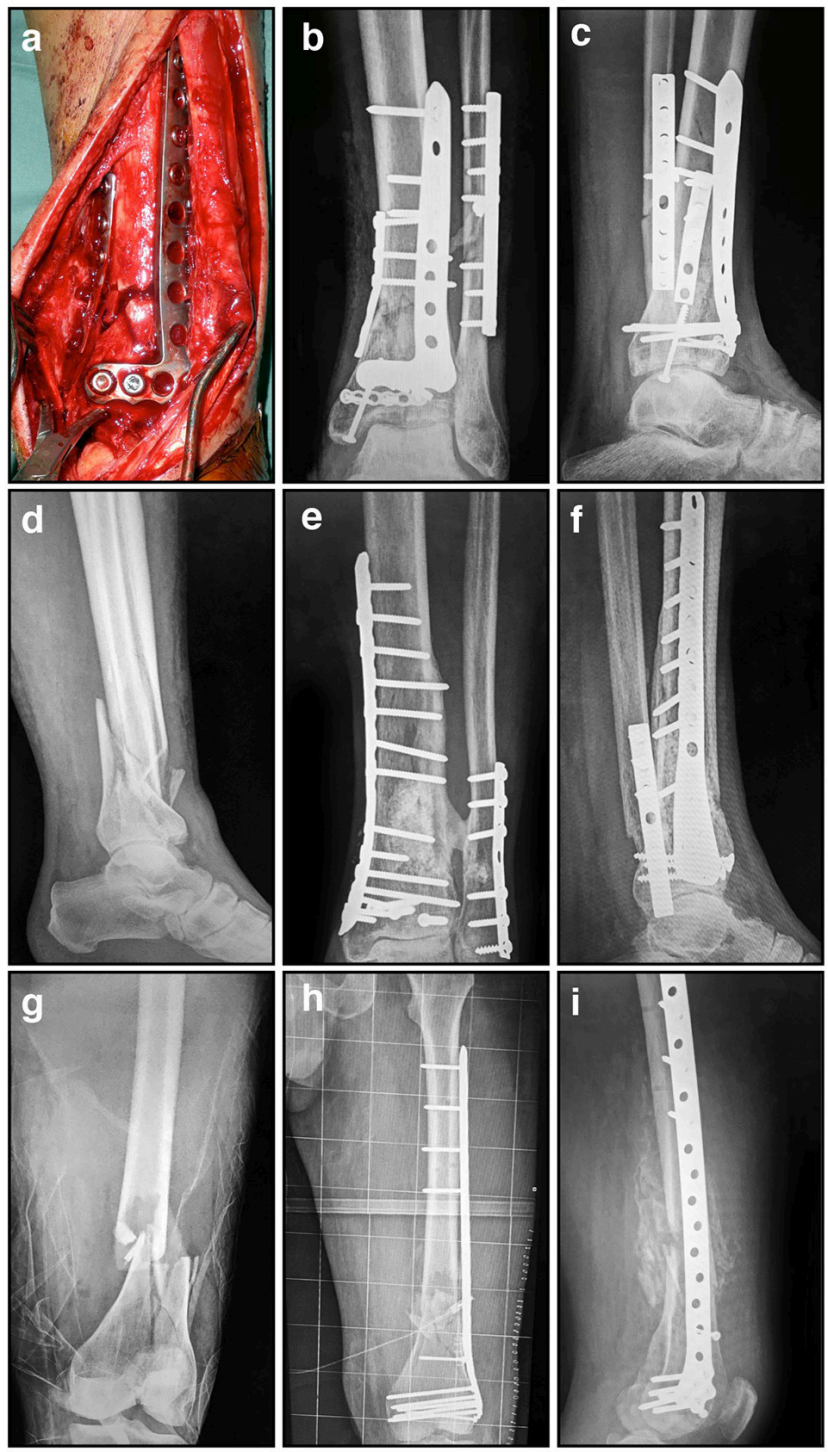
**Radiographic and intra-operative images illustrating internal fixation using plates: (A)** Intra-operative image showing distal tibial fracture fixation with two small plates; **(B)** AP radiograph showing distal tibial fracture fixation with two plates, and a single plate for the associated fibular fracture; **(C)** Corresponding lateral radiograph demonstrating stabilization using these three small plates; **(D)** Comminuted distal tibial/fibular fracture; **(E)** AP radiograph of relatively rigid distal tibial/fibula fracture fixation with a long medial locked plate; **(F)** Corresponding lateral radiograph of this distal tibial/fibular fracture fixation; **(G)** Comminuted distal femoral fracture; **(H)** AP radiograph of relatively flexible distal femoral fracture fixation with a lateral locked plate that bridges the zone of comminution; **(I)** Corresponding lateral radiograph of this distal femoral fracture stabilized with a lateral locked plate.

For instance, when a fracture has an open soft tissue wound or is potentially infected, external or internal fixation devices are more often used. Internal fixation of fractures dates back to the mid 1800s when Lister introduced the concept of ORIF for patella fractures. Since then, internal metal plates to stabilize fractures have been used for over 100 years. While internal fixation devices are available in various sizes and shapes for different bones and anatomical sites (Figure 3), initially, internal fixation methods were complicated by many hurdles such as infection, insufficient strength, poor surgical techniques, and corrosion, which were a consequence of limited biological and mechanical knowledge of fracture healing. Over the past several decades, advances in these sciences have significantly improved internal fixation designs and techniques, although some problems still exist. An ideal plate should accelerate fracture healing while not interfering with bone physiology. After their preliminary introduction, Lane (1895), Lambotte (1909), and Sherman (1912) subsequently abandoned metal internal fixation plates due to problems with corrosion and insufficient strength. Eggers (1948) then designed a plate that had two long slots, allowing the screw heads to slide thereby compensating for the resorption of the fragment ends. However, the use of this plate was limited and eventually also abandoned, because its structural weakness resulted in unstable fixation.

Danis first recognized the virtue of compression between the fracture fragments (Danis, 1949). He achieved this using an internal plate that suppressed IFM and increased fixation stability, resulting in primary bone healing. The compression plate underwent further design modifications when oval holes were created to provide interfragmentary compression during screw tightening, while another strategy achieves interfragmentary compression by tightening a tensioner that was temporarily anchored between the bone and plate. These design changes set the stage for the rigid plating of bone fractures, where healing advances without periosteal callus formation. In fact, any appearance of callus formation was thought to indicate fracture instability (Perren et al., 1988). The dynamic compression plate (DCP) was developed after improved designs to the rigid plate. The advantages of the DCP system were related to the low incidence of malunion, stable fixation, and the lack of any need for external immobilization. However, despite being superior to prior plate designs the DCP had some disadvantages, including delayed union and cortical bone loss under the plate. Furthermore, it was difficult to assess the progress of fracture healing radiologically due to the absence of callus formation. To avoid cortical porosity at the bone-plate interface, a new plate was designed called the limited contact-dynamic compression plate (LC-DCP), intended to have a 50% reduced contact area (Uhthoff et al., 2006). However, independent studies confirmed that the LC-DCP plating system was not advantageous in fracture healing, and did not restore cortical bone perfusion to the devascularized cortex. These studies agreed that cortical porosity was related to stress shielding induced by rigid fixation, and was not due to the remodeling of necrotic bone under the plate (Uhthoff and Dubuc, 1971; Akeson et al., 1976; Uhthoff et al., 1981, 1994; Uhthoff and Finnegan, 1983). Most importantly, the initial hypothesis stating that rigid fixation was necessary for fractures to heal uneventfully was abandoned with the introduction of biological osteosynthesis (Gerber et al., 1990). With this new concept the appearance of callus formation was actually a favorable sign suggesting rapid fracture healing, thereby refuting earlier statements that promoted rigid internal fixation. The desire to provide more biologically responsive stabilization then led to the development of point-contact fixation (PC-Fix), which eliminated interfragmentary compression and bicortical fixation. Unfortunately, a new plate design with very minimal plate-bone contact failed to prove beneficial clinically (Eijer et al., 2001; Haas et al., 2001; Uhthoff et al., 2006) and did not solve the problem of delayed union. However, this new plating system did improve fixation strength, especially for osteoporotic bone, and encouraged fracture healing through periosteal callus formation.

**Figure 4:**
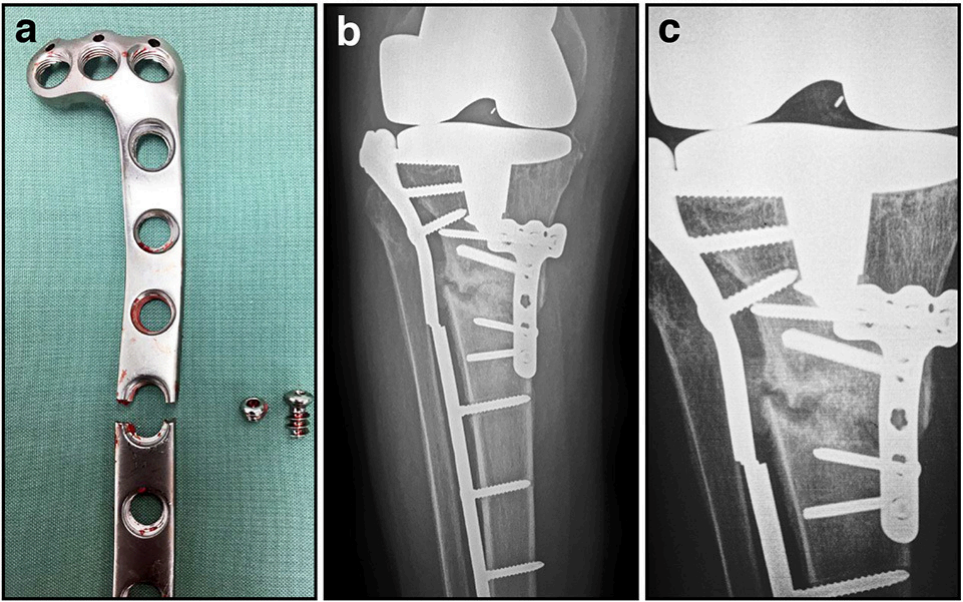
**Post-operative and radiographic images illustrating fatigue failure of an internal fixation locking plate. (A)** Appearance of broken locking plate having failed, as expected, through a screw hole; **(B)** AP radiograph showing proximal tibial non-union and failed implant; **(C)** Close-up of AP radiograph showing non-union and failed implant. The lateral plate has characteristically broken through a screw hole at the same level as the non-union.

The current school of thought for successful fracture healing using internal fixation adheres to two fundamental principles. The first is to achieve the optimal anchoring of the implant to the bone surface which maintains the fracture reduction during the healing process. The second involves simultaneously reducing the amount of soft tissue damage during the surgery, thereby maintaining local vascularity. To accomplish this the type of plate, the number of screws, and their positions should be carefully considered and must be suitable for each fracture situation. Rigid fixation with stiff compression plates following anatomical reduction is still an established method with excellent clinical results for many fractures (Figures 3D–F; Ruedi et al., 2007). However, when the fracture is highly comminuted and anatomic reduction would require extensive exposure that would devitalize the fragments, it is now considered preferable to employ “bridge” plating using less invasive techniques that preserve local vasculature (Chrisovitsinos et al., 1997; Farouk et al., 1998, 1999; Stoffel et al., 2003). This required the development of locked screw and plate designs, to achieve functional unity of the various elements of the construct. Locked plating and fixed-angle devices have become increasingly common over the past decade, and the altered mechanical environment can result in improvements in fracture healing when applied correctly in appropriate cases (Figures 3G–I; Gautier and Sommer, 2003; Stoffel et al., 2003). However, in some circumstances the constructs are considered too rigid and fracture healing can be delayed, resulting in implant failure and subsequent non-union (Figure 4; Gautier and Sommer, 2003; Lujan et al., 2010). Further research to address this issue has led to the development of modified designs that limit the stiffness of the implant-fracture construct, including far-cortical locking screws (Bottlang et al., 2009) and the recently proposed “active” plate (Bottlang et al., 2016). It is far too soon to know whether either of these developments will demonstrate sufficient advantages clinically, or will instead lead to further modifications to internal fixation.

### Intramedullary nails

**Figure 5:**
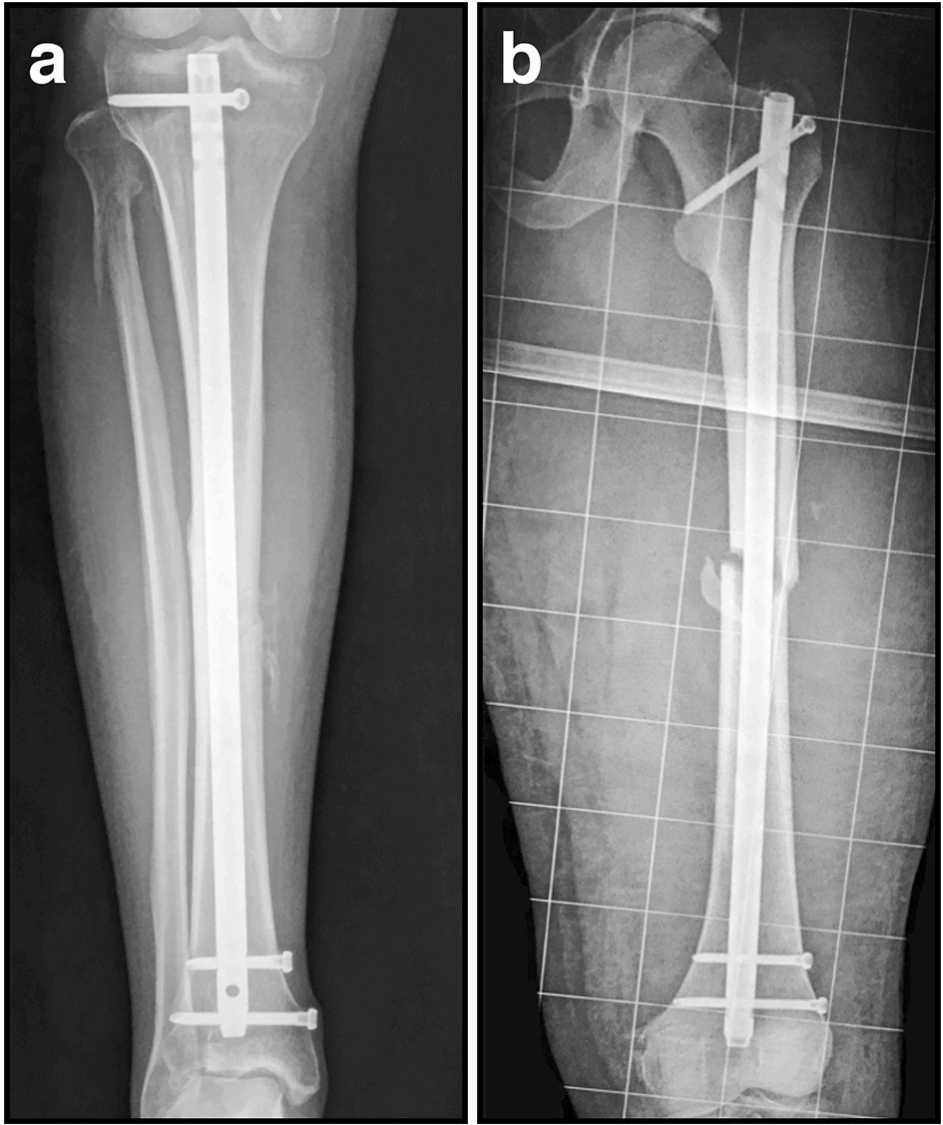
**Radiographic intramedullary nail images. (A)** Statically locked tibial nail to stabilize a mid-diaphyseal fracture; **(B)** Statically locked femoral nail to stabilize a mid-diaphyseal short oblique fracture.

The design of intramedullary nails has advanced considerably since the first attempts during ancient times using wooden dowels, and later with ivory pegs in the 1800's (Gluck, 1890; Konig, 1913; Hippocrates, 1938). In fact, autologous bone was even suggested and used as an intramedullary implant in an effort to fix fractured bone. This technique involved cutting out a section of cortical bone, which was then passed up the medullary canal across the fracture site (Hoglund, 1917). In 1939 Gerhard Kuntscher (Kuntscher, 1958), an orthopedic surgeon from Kiel, Germany, was primarily responsible for the development of the current day intramedullary nail. However, his method was not initially well received by fellow surgeons around the world, and it was considered an atrocity when it was discovered intramedullary nails were used to stabilize femur fractures in injured US pilots downed over Germany during World War II. Since its inception, this technique has been modified and applied with numerous improvements for the treatment of a diverse range of long bone fractures (Figure 5). Fracture healing following intramedullary nailing is very similar to what occurs during spontaneous, unsupported healing in nature, and as with cast or splint fixation, is accompanied by bridging callus formation.

The cross section of the first intramedullary nail was V-shaped, but was later modified to a cloverleaf pattern to improve resistance to torsional loads. In the 1950's, Lottes (1954, 1974) developed a flexible, unreamed, triflanged tibial nail, which was different from the cloverleaf shaped nail designed by Kuntscher (1958). This design was chosen to conform to the shape of the tibia, and gave it a markedly different flexibility and stiffness. In theory, at the time of insertion the nail would conform to the shape of the tibial medullary canal, and through elasticity would then revert to its original shape, while both reducing and stabilizing the fracture. Nevertheless, despite reports from a decade earlier noting an approximately 90% success rate was achieved using straight Kuntscher nails depending on the fracture configuration (Zucman and Maurer, 1969; d'Aubigne et al., 1974), perforation of the posterior tibial cortex was the main complication associated with straight Kuntschner nails. Herzog addressed this problem by adding a 20° apex posterior curve, with an additional 5° apex posterior curve of the distal nail (Donald and Seligson, 1983). These curves allowed easier insertion of the nail into the medullary canal, and this proximal curve remains an integral part of the design of contemporary nails. Although the nail design was improved by adding curvature, fracture outcomes were not significantly improved. A report by d'Aubigne et al. (1974) suggested that tibial fracture stabilization with intramedullary nailing offered poor fixation at the upper and lower ends of the bone. Grosse et al. (1978) later added interlocking screws that could be inserted through the nail and the bone on both sides of the fracture. The use of interlocking screws prevented rotational movement and telescoping (Figure 5), which improved fracture stability and allowed for earlier weight bearing (Kempf et al., 1985). Furthermore, interlocking nails had either dynamic holes allowing fracture compression during weight bearing, or static holes providing greater stability without allowing fracture compression. Since then the main design elements of nails have not changed significantly, although different insertion approaches have been used to improve surgical outcomes (Tornetta and Collins, 1996; Court-Brown et al., 1997; Keating et al., 1997; Tornetta et al., 1999; Toivanen et al., 2002). Titanium was introduced as an alternative because its material properties more closely replicate the modulus of elasticity of normal bone, in an effort to promote more uniform and rapid union. However, the transition from stainless steel to titanium nails over the past two decades has resulted in implants with thicker walls that behave nearly the same biomechanically, and with negligible clinical benefits (Bong et al., 2007). Before a nail is inserted, the medullary canal is generally reamed to allow a larger nail to be used, and to improve the fit between the surface of the nail itself and endosteal bone, which maximizes contact and limits instability that might eventually result in implant failure (Bong et al., 2007). Although a large prospective randomized clinical study demonstrated reamed nailing was mildly superior to unreamed nailing for closed tibial shaft fractures, this was not true for open fractures where reaming provided no apparent benefit (Bhandari et al., 2008).

**Figure 6:**
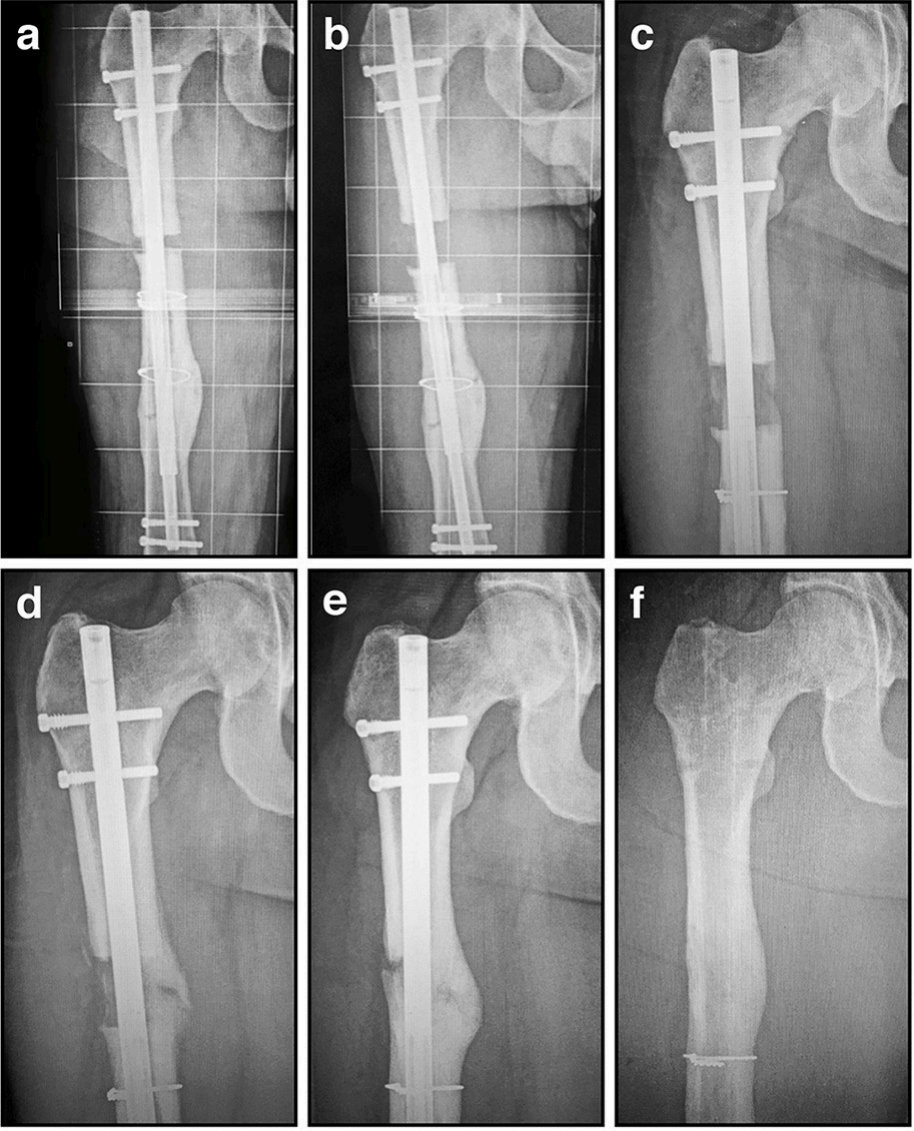
**Radiographic images illustrating the process of gradual femoral lengthening using a telescopic nail: (A)** AP radiograph of a femoral lengthening nail obtained early post-operative, following only limited distraction; **(B)** AP radiograph of a telescopic lengthening nail after completing distraction of 2.7 cm; **(C)** Radiographic image illustrating lengthening gap at 6 weeks, demonstrating early regenerate bone formation; **(D)** Radiographic image at 12 weeks, as the regenerate gradually matures and consolidates; **(E)** Radiographic image illustrating lengthening gap at 24 weeks, as the regenerate bone matures further and hypertrophies; **(F)** Radiographic image illustrating fully consolidated gap at 52 weeks, following removal of the telescopic nail.

Typical intramedullary nails are passive devices with biomechanical advantages that encourage the spontaneous union of diaphyseal fractures. However, nail technology became much more sophisticated when these devices were introduced as active elements to achieve limb lengthening by Bliskunov (1983). Distraction histogenesis is most commonly employed for limb length equalization and deformity correction, or when lengthening for stature. As remarkable as this process is, it has become commonplace in the realm of limb lengthening and reconstruction (Ilizarov, 1989a,b; Fischgrund et al., 1994; Tetsworth and Paley, 1995). Lengthening nails are generally telescopic in design (Figure 6), with a slightly smaller tube gradually extruded from within a slightly larger tube. The various implants available use different strategies to provide the necessary distraction force, including mechanical, motorized, or magnetic devices (Betz et al., 1990; Baumgart et al., 1997; Cole et al., 2001; Thaller et al., 2014; Kucukkaya et al., 2015; Paley, 2015; Paley et al., 2015). These adjustable intrameduallary nails are a less invasive alternative to external fixators, and are therefore more attractive to both patients and surgeons. Yet both types of device achieve exactly the same objective, controlled gradual mechanical distraction to produce bone growth when desired (Figures 6A–F; Hasler and Krieg, 2012).

The latest iteration of these implants relies on an external rotating magnet inducing motion in an internal magnet, that in turn drives a gearbox to achieve lengthening (Thaller et al., 2014; Kucukkaya et al., 2015; Paley, 2015; Paley et al., 2015). This technology was originally introduced by Verkerke in 1989, for use in an expandable endoprosthesis following tumor resection in pediatric patients (Verkerke et al., 1989). Powered devices, either motorized or magnetically driven, offer unprecedented control over the implant, and provide the ability to modulate the process of distraction and directly influence the growth of bone (Betz et al., 1990; Thaller et al., 2014; Kucukkaya et al., 2015; Paley, 2015; Paley et al., 2015). It is now possible to increase or decrease the rate of distraction, and to discontinue or even reverse the process if necessary. Gaining popularity at this time, magnetically powered lengthening nails have been successfully employed in over 1000 cases globally (Paley, 2015). Fully implanted distraction devices like these decrease the risk of pin site infection associated with external fixation, but lengthening still exposes the patient to the other associated risks of nerve injury, contracture, subluxation, or secondary deformity (Thaller et al., 2014; Kucukkaya et al., 2015; Paley, 2015; Paley et al., 2015).

### External fixators

**Figure 7:**
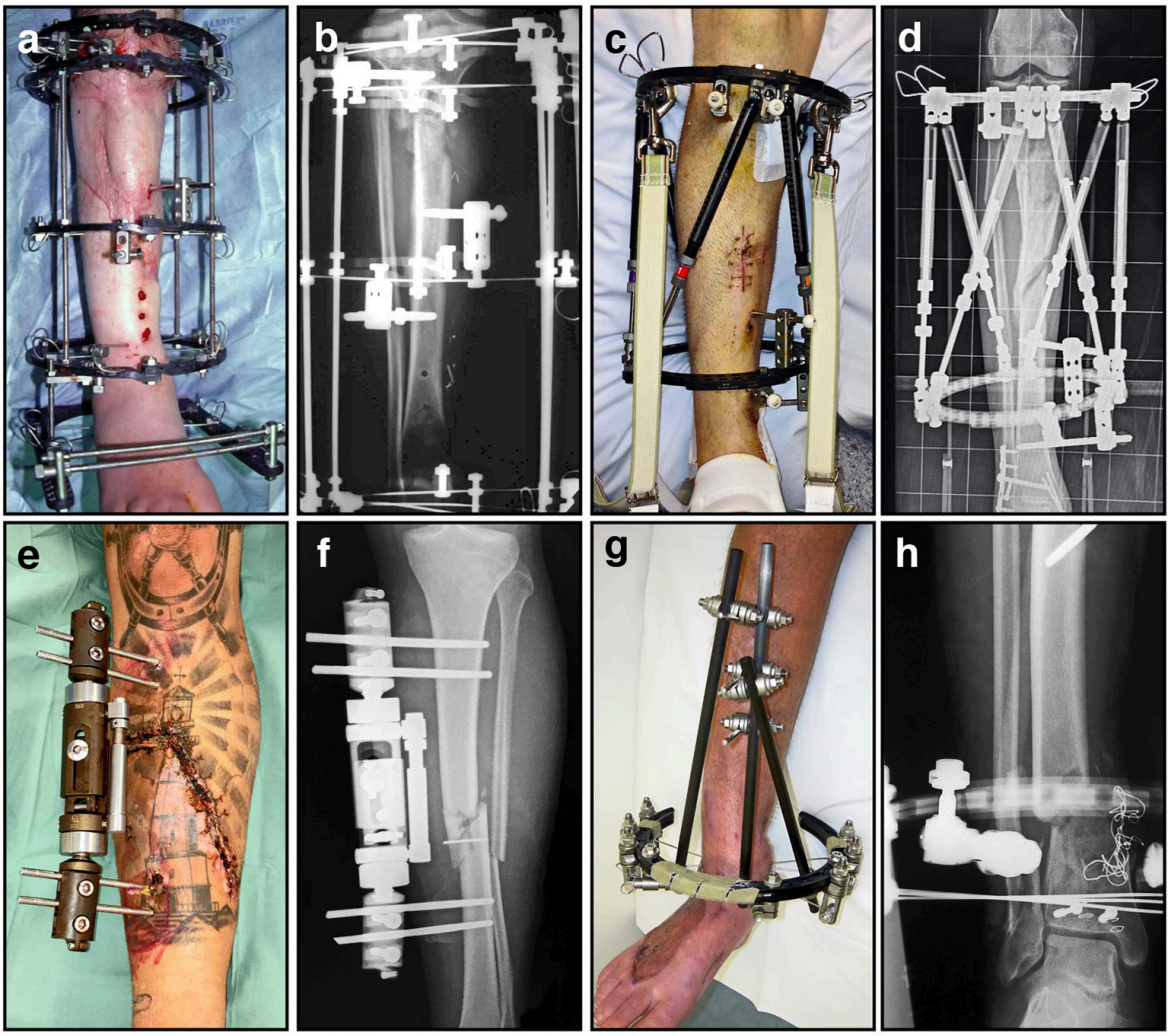
**A variety of external fixators are used for fracture fixation, most often for tibia fractures as demonstrated here. (A)** Clinical image of an Ilizarov external fixator applied to a fractured leg, with multiple rings and tensioned wires; **(B)** Corresponding radiograph illustrating proximal tibial fracture fixation with this circular frame; **(C)** Clinical image of a hexapod-based external fixator applied to a fractured limb, using a pair of rings; **(D)** Corresponding radiograph illustrating stabilization of a comminuted segmental tibia fracture; **(E)** Clinical image of a unilateral fixator in position; **(F)** Corresponding radiograph demonstrating mid-tibial open fracture fixation using this device; **(G)** Clinical image of a hybrid (cantilever) external fixator, incorporating a single juxta-articular ring and unilateral diaphyseal elements; **(H)** Corresponding radiograph demonstrating a distal tibial fracture stabilized using this device.

Despite the inconvenience to the patient and the high likelihood of superficial pin site infections, external fixators fill a clinical niche when other methods of fracture stabilization are unsuitable. Following high energy trauma with open injuries, plates and intramedullary nails are sometimes considered an unacceptable risk for deep infection. Occasionally the extent of comminution and the breadth of involvement results in a fracture configuration inherently unstable, but external fixators can even span joints to better control the fracture mechanically (Figure 7). Significantly, unlike plates and most intramedullary nails, external fixators provide opportunites for postoperative modification. The adjustability of external fixators has, until recently, been unique, and in part explains why they continue to play an important role in musculoskeletal trauma care. External fixation has transitioned from being a last resort fixation method to becoming the method of choice when treating some of the most challenging bone pathology encountered clinically. Although alternative treatment strategies are available, it continues to be an essential element in limb salvage during both early and late bone reconstruction of serious extremity injuries. It is currently the only system that allows the surgeon to control the flexibility of the fixation during the course of bone healing. External fixators have evolved dramatically from the most primitive designs incorporating wooden splints, to contemporary designs where a wide array of metals and composite materials are used. Unfortunately, with the evolution of these devices came many complications, and it has become a more technically demanding procedure. Despite these factors, many surgeons worldwide continue to use external fixators to treat complex fractures, segmental defects, and congenital deformities.

The work of many clinicians, researchers, and engineers from around the world are responsible for the current design of external fixation devices. For example, the distraction and compression mechanisms of modern devices are credited to Lambret from 1911 (LaBianco et al., 2001). In 1931 Pitkin and Blackfield were the first surgeons to advocate bi-cortical pins attached to two external fixation clamps, as a bilateral frame to improve fracture healing. Anderson et al. presented a series of papers from 1933–1945 (Anderson, 1935, 1938; Anderson and Burgess, 1943), outlining the use of both half-pins and transfixation pins for the treatment of various long bone fractures, arthrodesis, and limb lengthening procedures (Christian, 1998). These gradual incremental improvements have resulted in the designs currently available today, providing external fixators that come in three major configurations (Figure 7): circular (A–D), monorail (unilateral) (E–F) and hybrid (G–H).

**Figure 8:**
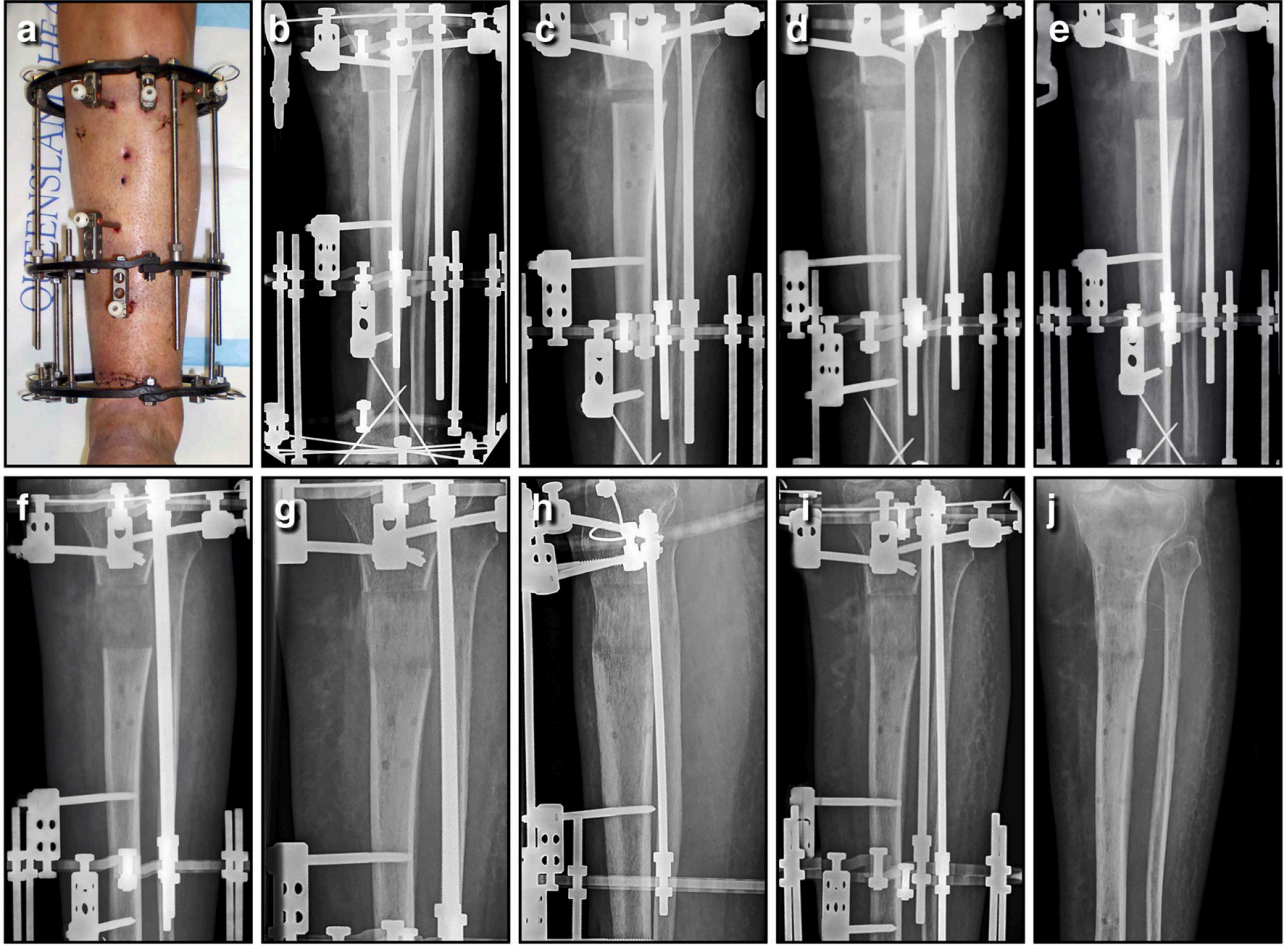
**Bone lengthening can be achieved through distraction osteogenesis using an Ilizarov external fixator**. In this instance, an open distal tibial fracture resulted in a 3.7 cm segmental defect; the limb was acutely shortened, the wound closed primarily, and length gradually restored through a proximal corticotomy. This panel features a series of AP tibial radiographs demonstrating the 6 month process, and the final result a full year later; **(A)** Clinical image of the fractured limb stabilized with a circular frame after acute shortening of 3.7 cm; **(B)** AP radiograph immediately post-operative, with the proximal corticotomy minimally displaced; **(C)** AP radiograph of proximal tibia lengthening after 2 weeks of distraction; **(D)** AP radiograph of a proximal tibia lengthening at 4 weeks, with regenerate bone visible in the distraction gap; **(E)** AP radiograph of a proximal tibia lengthening at 6 weeks, as the gap slowly increases in size; **(F)** AP radiograph of a proximal tibia lengthening at 8 weeks, after length has been restored; **(G)** AP radiograph of a proximal tibia lengthening at 12 weeks, as the new bone gradually matures; **(H)** AP radiograph of a proximal tibia lengthening at 18 weeks, as the bone continues to consolidate; **(I)** AP radiograph of a proximal tibia lengthening at 26 weeks, shortly before the regenerate bone was solid enough to allow removal of the frame; **(J)** AP radiograph of the lengthened proximal tibia at 52 weeks, as the bone continues to strengthen and remodel. This regenerate bone will eventually become indistinguishable from surrounding normal bone.

Professor Gavril A. Ilizarov must be acknowledged not only for his contributions to the modern design of unilateral and circular external fixators, but also for inventing limb salvage and bone lengthening procedures through distraction osteogenesis (Ilizarov, 1988; Ilizarov and Frankel, 1988). Clinically, distraction osteogenesis is the perfect example of using a fixation device to apply mechanical forces to stimulate the process of bone regeneration. This is achieved with a specialized form of external fixation known as a ring or circular fixator (Figures 7A,B). Ilizarov found these external frames invaluable for multiple applications including post-traumatic and congenital limb reconstruction, management of osteomyelitis, regeneration of bone defects, deformity correction, and complex arthrodesis. These devices utilize Ilizarov's principle of distraction histogenesis, and rely on a special type of low energy osteotomy that preserves local vasculature (Ilizarov, 1989b). Ideally, only the cortical bone is fractured, leaving the medullary vessels and the periosteum intact in the metaphyseal region. After an initial latency period to allow the osteotomy to begin to heal, the fixator is adjusted on a regular basis to achieve controlled gradual mechanical distraction (Ilizarov, 1989a). As the fixator is slowly lengthened, new bone forms in the gap created at the osteotomy by the now familiar process of distraction osteogenesis (Ilizarov, 1989a,b). For example, this proces allows for skeletal reconstruction across segmental defects through bone transport, using small tensioned Kirschner wires (K-wires) and circumferential ring supports (Figure 8). As new bone growth occurs in the metaphyseal region, a segment of healthy bone is gradually translocated into the defect. The tension that is created by gradual mechanical distraction stimulates the formation of new bone, skin, blood vessels, peripheral nerves, and muscle during the analogous process of distraction histogenesis (Figures 8C–J). Through this remarkable process, bone lengthening and regeneration can occur at a rate of approximately one centimeter per month. Ilizarov techniques have come a long way in treating non-unions by the mechanical stimulation and modulation of callus for the reconstruction of segmental defects well in excess of what iliac crest bone graft can reliably fill. More importantly, this has resulted in this technique giving rise to limb salvage having the superior quality of regenerated normal bone.

The basic components of the circular frame are rings, tensioned wires, and connecting threaded rods. The stability of the frame depends upon the configuration of the basic components (Calhoun et al., 1992; Kummer, 1992; Podolsky and Chao, 1993; Lenarz et al., 2008), and this will influence the local mechanical environment around the regenerated bone, thereby determining the type, rate, and quality of the tissue formed. For instance, depending on the type and size of the rings (full, partial, or arches) the stability of the construct will change. Full rings provide the most stability, partial intermediate, and arches the least. The diameter of the rings is also very important, and smaller rings are inherently more stable than larger ones of the same thickness (Calhoun et al., 1992; Kummer, 1992; Podolsky and Chao, 1993; Lenarz et al., 2008). Frame stability will also be dependent upon the distance between rings, and the type and quantity of ring connectors such as wires, rods, and Shantz pins. In clinical practice, various combinations of the circular frame components are used, depending on the intended application and required stability.

## Future perspectives

Bone has an amazing ability to heal spontaneously without forming scar tissue. When bone fails to heal, from either a large defect or non-union of a fracture, it is necessary to consider morphogenetic signals, cells, scaffolds and the precise mechanical conditions in order to achieve successful union. This review, focusing on the last of these, has summarized the decades of work and acquired knowledge that has been used to develop new treatments and technologies for the regeneration of bone. Research has now shown how the precise mechanical environment provided by the fixation stability around the bone lesion significantly influences the way bone heals. Future convergence of this research with the findings of complementary studies in biology, tissue engineering, and regenerative medicine promises important synergies. Bringing together these diverse disciplines in a productive manner remains a challenge for the future.

An important goal of mechano-biology is to determine which biological signals can be successfully manipulated through modulating the local mechanical environment, for instance, by adjusting fixation stability. Identifying these molecular signals will allow them to be targetted in novel ways to initiate, maintain, and accelerate the repair process. Unraveling the interplay between mechanics and biology in this setting is a problem for the emerging field of systems biology (Bizzarri et al., 2013; Giorgi et al., 2016). The levels and types of complexity are massive, and their understanding will require a big data approach. Moreover, we currently lack the experimental tools for a comprehensive investigation of the problem. Although *in vitro* experiments can be performed with sophistication, *in vivo* studies lack the tools for precise, controlled experiments. In particular, there is a need for non-invasive sensors that permit controlled, spatially defined, real-time analysis of the chemical, physical, and biological environment within defects as they heal. Such deficiencies are compounded by the lack of sophisticated ways to control the mechanical environment of a healing defect with precision at a cellular level. Contemporary plates, fixators, and rods are clumsy and imprecise in this regard. Furthermore, the informed and appropriate selection of scaffolds and growth factors for specific purposes, and insights into how these factors interact with mechanical cues, will require additional research.

Finally, there is a need to incorporate this new information into clinical practice. Little consideration of mechanical factors is often given when choosing the type of fixation device to stabilize a fracture. Compounding this deficit is the lack of *in vivo* monitoring tools with which to inform decisions of this type. New methods of non-invasive monitoring of bone healing are being developed that will allow us to address this issue. These should allow for the selection of the appropriate fixation device for any given circumstance, optimize decisions concerning the removal of the device, and encourage an early return to normal weight bearing.

## Author contributions

All authors listed have made substantial and direct intellectual contribution to the work, and approved it for publication.

### Conflict of interest statement

The authors declare that the research was conducted in the absence of any commercial or financial relationships that could be construed as a potential conflict of interest. The reviewer CC and handling Editor declared their shared affiliation, and the handling Editor states that the process nevertheless met the standards of a fair and objective review.
